# How Phytohormones Shape Interactions between Plants and the Soil-Borne Fungus *Fusarium oxysporum*

**DOI:** 10.3389/fpls.2016.00170

**Published:** 2016-02-16

**Authors:** Xiaotang Di, Frank L. W. Takken, Nico Tintor

**Affiliations:** Molecular Plant Pathology, Faculty of Science, Swammerdam Institute for Life Sciences, University of AmsterdamAmsterdam, Netherlands

**Keywords:** plant immunity, root pathogen, vascular wilt disease, effectors, endophyte

## Abstract

Plants interact with a huge variety of soil microbes, ranging from pathogenic to mutualistic. The *Fusarium oxysporum* (*Fo*) species complex consists of ubiquitous soil inhabiting fungi that can infect and cause disease in over 120 different plant species including tomato, banana, cotton, and Arabidopsis. However, in many cases *Fo* colonization remains symptomless or even has beneficial effects on plant growth and/or stress tolerance. Also in pathogenic interactions a lengthy asymptomatic phase usually precedes disease development. All this indicates a sophisticated and fine-tuned interaction between *Fo* and its host. The molecular mechanisms underlying this balance are poorly understood. Plant hormone signaling networks emerge as key regulators of plant-microbe interactions in general. In this review we summarize the effects of the major phytohormones on the interaction between *Fo* and its diverse hosts. Generally, Salicylic Acid (SA) signaling reduces plant susceptibility, whereas Jasmonic Acid (JA), Ethylene (ET), Abscisic Acid (ABA), and auxin have complex effects, and are potentially hijacked by *Fo* for host manipulation. Finally, we discuss how plant hormones and *Fo* effectors balance the interaction from beneficial to pathogenic and *vice versa*.

## Introduction

*Fusarium oxysporum* (*Fo*), one of the most relevant plant pathogens in global agriculture, is a widespread soil-borne fungus that invades roots and causes vascular wilt disease through colonization of xylem tissue (Tjamos and Beckman, 1989). Pathogenic *Fo* strains have been classified in more than 120 *formae speciales* (ff.spp.), which refers to a specific plant host, as a particular isolate typically produces disease only within a limited range of host species (Armstrong and Armstrong, 1981; Katan and Di Primo, 1999; Table 1). The infection process occurs following attachment to the root surface and subsequent penetration and colonization of the plant root and proliferation within the xylem vessels, leading to both local and systemic induction of a broad spectrum of plant defense responses (Berrocal-Lobo and Molina, 2008). Vascular browning, stunting, progressive wilting, and eventually plant death are typical disease symptoms in infected plants (Pietro et al., 2003; Agrios, 2005). In contrast to the potential of pathogenic *Fo* isolates to cause destructive plant diseases, many *Fo* strains are non-pathogenic and survive either saprophytically in the soil, as non-invasive colonizer of the rhizosphere, or as endophyte inside plant tissues (Kuldau and Yates, 2000; Edel-Hermann et al., 2015; Imazaki and Kadota, 2015). There is relatively little known about the lifestyle strategies of these inconspicuous endophytic strains, but some of them have been successfully employed in biocontrol strategies to combat plant diseases (Alabouvette et al., 2009; Vos et al., 2014).

**Table 1:** ***Fo* strains and their host described in the manuscript**.

**Abbreviations**	***Formae speciales***	**Host**
*Foal*	*Fusarium oxysporum* f.sp. *albedinis*	Date palm
*Focn*	*Fusarium oxysporum* f.sp. *conglutinans*	Cabbage, Arabidopsis
*Focb*	*Fusarium oxysporum* f.sp. *cubense*	Banana
*Fol*	*Fusarium oxysporum* f.sp. *lycopersici*	Tomato
*Fomt*	*Fusarium oxysporum* f.sp. *matthioli*	Garden stock, Arabidopsis
*Foph*	*Fusarium oxysporum* f.sp. *phaseoli*	Common bean
*Forp*	*Fusarium oxysporum* f.sp. *raphani*	Radish, Arabidopsis
*Forl*	*Fusarium oxysporum* f.sp. *radicis-lycopersici*	Tomato

Based on their lifestyle plant pathogenic fungi have been classified as biotrophs and necrotrophs. Biotrophic pathogens derive nutrients from living cells and deploy complex manipulation strategies to exploit their hosts while keeping them alive. In contrast, necrotrophic pathogens generally kill host cells and feed on their contents, resulting in extensive necrosis, tissue maceration, and plant rot (Glazebrook, 2005). A third type, termed hemi-biotrophs, displays both forms of nutrient acquisition, shifting from a biotrophic phase early in infection to necrotrophy at later stages. These pathogens typically produce toxins only at later stages of disease development in order to kill the host cells and to complete their life cycle on dead tissues (Horbach et al., 2011). The strategy of different pathogenic *Fo* strains can vary, but is usually best described by a hemi-biotrophic lifestyle (Michielse and Rep, 2009). Consistently, the *Fo* genomes show an expansion of genes that encode small, secreted proteins as well as cell-wall degrading enzymes, a feature shared by many hemi-biotrophic fungi (Lo Presti et al., 2015). Analysis of the xylem sap proteome from *Fol-*infected tomato plants identified numerous fungal proteins, termed Secreted in Xylem (Six) protein. For several Six proteins a contribution to virulence has been demonstrated, designating them as *bona fide* effectors (Takken and Rep, 2010; de Sain and Rep, 2015). However, their molecular mode of action and putative virulence targets remain unknown.

Phytohormones such as SA, JA, and ET, are known to play major roles in regulating plant defense responses against various pathogens. Generally, SA signaling triggers resistance against biotrophic and hemibiotrophic pathogens, whereas a combination of JA and ET signaling activates resistance against necrotrophic pathogens (Glazebrook, 2005). All these hormones are part of a larger signaling network that integrates environmental inputs and provides robustness against microbial manipulations (Katagiri and Tsuda, 2010; Pieterse et al., 2012). Additional hormones such as auxins, abscisic acid (ABA), gibberellic acids (GAs), and brassinosteroids (BRs) have also been reported to be involved in plant immunity and to fine-tune immunity and growth/development in plants (Table 2; Robert-Seilaniantz et al., 2011). In this review, we summarize the current knowledge on the role of phytohormones in plant disease and resistance triggered by different *Fo* ff.spp. to uncover how they shape the outcome of this widespread plant-fungal interaction.

**Table 2:** **Phytohormone mutants involved in the defense response against *Fo* infection**.

**Hormones**	**Mutants and transgenic lines**	**Process affected**	**Plant species**	**Susceptibility**	**References**
SA	*NahG*	SA accumulation	Arabidopsis	Increased to *Focn* and *Fol*	Berrocal-Lobo and Molina, 2004; Diener and Ausubel, 2005; Thatcher et al., 2009; Trusov et al., 2009
	*sid2-1*	SA biosynthesis	Arabidopsis	Increased to *Focn* and *Fol*	Berrocal-Lobo and Molina, 2004; Diener and Ausubel, 2005
	*eds1-1, eds1-22*	SA signaling	Arabidopsis	Unaltered to *Focn* and *Fol*	Berrocal-Lobo and Molina, 2004; Trusov et al., 2009
	*eds3, eds4, eds10*	SA signaling	Arabidopsis	Increased to *Focn*	Diener and Ausubel, 2005
	*eds5-1*	SA biosynthesis	Arabidopsis	Increased to *Focn* and *Fol*	Berrocal-Lobo and Molina, 2004; Diener and Ausubel, 2005; Thatcher et al., 2009; Trusov et al., 2009
	*pad4-1*	SA signaling	Arabidopsis	Unaltered to *Focn* and *Fol*	Berrocal-Lobo and Molina, 2004
	*pad4*			Increased to *Focn*	Diener and Ausubel, 2005
	*npr1-1*	SA perception	Arabidopsis	Increased to *Focn* and *Fol*	Berrocal-Lobo and Molina, 2004
	*npr1-1, npr1-2, npr1-3, npr1-4*	SA perception	Arabidopsis	Unaltered to *Focn*	Diener and Ausubel, 2005; Trusov et al., 2009
	*35S::NPR1*	SA perception	Tomato	Reduced to *Fol*	Lin et al., 2004
	*hpSAMT*	SA metabolism	Tomato	Reduced to *Fol*	Ament et al., 2010
JA	*aos, fad3-2, opr3 fad7-1 fad8*	JA biosynthesis	Arabidopsis	Unaltered to *Focn*	Thatcher et al., 2009
	*coi1, coi1-21*	JA perception	Arabidopsis	Reduced to *Focn* and *Fomt*,	Thatcher et al., 2009; Trusov et al., 2009; Cole et al., 2014
	*jar1-1*	JA-Ile biosynthesis	Arabidopsis	Increased to *Focn* and *Fol*	Berrocal-Lobo and Molina, 2004; Trusov et al., 2009
	*jar1-1*			Unaltered to *Focn*	Thatcher et al., 2009
	*jin1-9(atmyc2-3), jin1-9/myc2*	JA signaling	Arabidopsis	Reduced to *Focn*	Anderson et al., 2004; Trusov et al., 2009
	*35S::AtERF2*	Positive regulator of MeJA response	Arabidopsis	Reduced to *Focn*	McGrath et al., 2005
	*35S::AtERF4*	Negative regulator of MeJA response	Arabidopsis	Increased to *Focn*	McGrath et al., 2005
	*pft1-1, med8*	JA signaling	Arabidopsis	Reduced to *Focn*	Kidd et al., 2009
	*def1*	JA biosynthesis	Tomato	Increased to *Forl* and *Fol*	Thaler et al., 2004; Kavroulakis et al., 2007
	*jai1*	JA perception (Coi1 homolog)	Tomato	Unaltered to *Fol*	Cole et al., 2014
ET	*ein2-1*	ET signaling	Arabidopsis	Reduced to *Focn* and *Forp*	Trusov et al., 2009; Cole et al., 2014
	*ein2, etr1*	ET signaling	Arabidopsis	Unaltered to *Focn*	Thatcher et al., 2009
	*ein2-5*	ET signaling	Arabidopsis	Increased to *Focn* and *Fol*	Berrocal-Lobo and Molina, 2004
	*etr1-1*	ET perception	Arabidopsis	Reduced to *Forp*	Pantelides et al., 2013
	*35S::ERF1*	ET signaling	Arabidopsis	Reduced to *Focn* and *Fol*	Berrocal-Lobo and Molina, 2004
	*Never ripe*	ET perception	Tomato	Reduced to *Fol*	Lund et al., 1998; Francia et al., 2007
	*Never ripe, epinastic (epi1)*	ET signaling	Tomato	Unaltered to *Forl*	Kavroulakis et al., 2007
ABA	*aba1-6, aba2-1*	ABA biosynthesis	Arabidopsis	Reduced to *Focn*	Anderson et al., 2004; Trusov et al., 2009
	*aba2*	ABA biosynthesis	Arabidopsis	Reduced to *Focn*	Cole et al., 2014
Auxin	*cyp79b2 cyp79b3, atr4/sur2, myb51/hig1, atr1, atr2d, pad3*	auxin biosynthesis	Arabidopsis	Unaltered to *Focn*	Kidd et al., 2011
	*35S:ATR1**/**MYB34, atr1d, 35S:ATR2*				
	*axr1, axr2, axr3, sgt1b*	auxin signaling	Arabidopsis	Reduced to *Focn*	Kidd et al., 2011
	*tir1*	auxin perception	Arabidopsis	Unaltered to *Focn*	Kidd et al., 2011
	*arf1, arf2, arf1arf2*	auxin signaling	Arabidopsis	Reduced to *Focn*	Lyons et al., 2015

## SA promotes resistance to *Fo*

Defense to biotrophic or hemibiotrophic pathogens is frequently mediated via SA signaling (Glazebrook, 2005). Arabidopsis plants with impaired SA accumulation showed increased susceptibility to *Fo* f.sp. *conglutinans* (*Focn*), but not to *Fo* f.sp. *raphani* (*Forp*) pointing to an isolate-specific role of SA-dependent defense responses (Table 2; Berrocal-Lobo and Molina, 2004; Diener and Ausubel, 2005; Trusov et al., 2009; Cole et al., 2014). Interestingly, mutants of the SA master-signaling regulator NPR1 showed wildtype (WT)-like susceptibility to *Focn* when 2–3-week-old soil grown plants were examined (Diener and Ausubel, 2005; Trusov et al., 2009). In contrast, when seedlings were infected on sterile agar plates *npr1* mutants displayed clearly enhanced susceptibility in comparison to WT (Berrocal-Lobo and Molina, 2004). SA accumulation and signaling is also influenced by the nucleo-cytoplasmic proteins PAD4 and EDS1. Soil-grown *pad4*, but not *eds1*, Arabidopsis showed increased susceptibility, whereas sterile grown *pad4* seedlings behaved like WT (Berrocal-Lobo and Molina, 2004; Diener and Ausubel, 2005; Trusov et al., 2009). Thus, the influence of SA signaling regulators depends on growth and inoculation conditions, plant age and the *Fo* isolate used. It appears that at least in soil-grown plants, SA enhances immunity to *Fo* via NPR1- and EDS1-independent pathways.

Consistent with its defense promoting role in Arabidopsis, exogenous application of SA, or synthetic SA analogs reduced *Fo* disease symptoms in a broad range of tested plants including tomato, common bean, date palm, and Arabidopsis (Edgar et al., 2006; Mandal et al., 2009; Dihazi et al., 2011; Xue et al., 2014). Furthermore, stable overexpression of Arabidopsis *NPR1* in tomato reduced disease symptoms as well as *Fo* f.sp. *lycopersici* (*Fol*) colonization of the stem (Lin et al., 2004). Similarly, preventing SA volatilization by silencing of a *Salicylic Acid Methyltransferase* reduced tomato susceptibility to *Fol*, however without significantly changing overall SA levels (Ament et al., 2010). How exactly Methyl-SA levels influence tomato defense to *Fol* remains to be addressed.

Despite a clear effect of SA on disease severity, global transcriptome profiling of Arabidopsis plants inoculated with *Focn* revealed relatively mild changes in the expression of known SA marker genes (Kidd et al., 2011; Zhu et al., 2013; Chen et al., 2014; Lyons et al., 2015). In fact, expression of *PR1* was even slightly down regulated both in roots and shoots of inoculated plants (Kidd et al., 2011). It is possible that activation of SA signaling occurs at rather late stages, which would be missed by the present studies that focus on the time points 1–6 days-post-inoculation (dpi). Alternatively, SA signaling could activate previously uncharacterized defense mechanisms, in line with the observed NPR1- and EDS1-independency, especially in roots that are still little explored in terms of plant immunity (De Coninck et al., 2015). A third possibility has been suggested by Cole et al.: SA signaling could serve to dampen activation of JA responses that are promoting Arabidopsis infection by *Fo* (see below; Cole et al., 2014). Similar to Arabidopsis, transcriptome profiling of banana saplings infected with virulent and avirulent *Fo* f.sp. *cubense* (*Focb*) strains also failed to detect activation of typical SA marker genes, at least at the relatively early time points analyzed (Li et al., 2013). This led to the suggestion that in banana defense against *Fo* is mainly mediated via ET/JA signaling (Swarupa et al., 2014).

**Figure 1:**
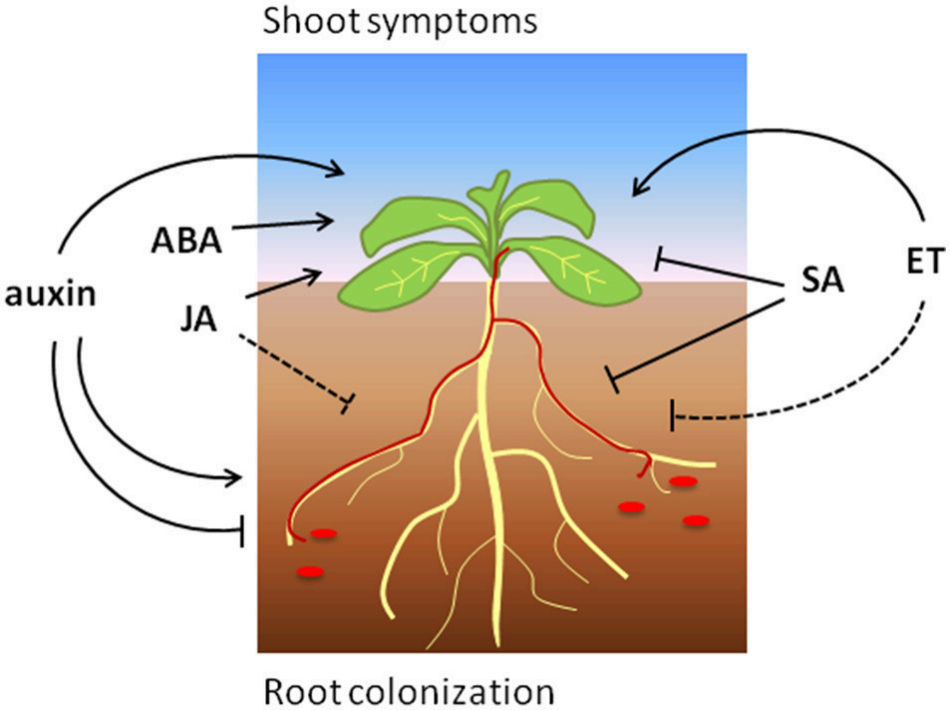
**The effects of phytohormone signaling on Arabidopsis root colonization and shoot symptoms induced by *Focn* (shown in red)**. Arrows and blunt ends indicate promotion and reduction, respectively. The dashed line indicates a presumed positive role of ET-signaling on (root) defense.

Taken together, SA signaling positively regulates defense to *Fo* in most tested plant species, which is in line with a predominantly hemi-biotrophic lifestyle of this pathogen (Figure 1). However, the exact mechanisms by which SA reduces susceptibility to *Fo* are not understood and yet unknown SA targets possibly play a role during defense to this root-infecting pathogen.

## JA signaling can promote either resistance or susceptibility in different host–*Fo* interactions

JA signaling generally mediates resistance to necrotrophic pathogens and insect herbivores. These two functions are exerted by two separate and often mutually antagonistic branches: the former is regulated by ERF transcription factors and is associated with ET signaling, whereas the latter requires MYC transcription factors and often involves ABA signaling (Pieterse et al., 2012).

In Arabidopsis JA biosynthesis is not critical for defense to *Focn* as a whole series of mutants with impaired JA accumulation behaved like WT plants (Table 2; Thatcher et al., 2009). Similarly, *jar1* mutants that are defective in synthesis of the bioactive JA–Isoleucine conjugate showed WT-like or slightly increased susceptibility (Thatcher et al., 2009; Trusov et al., 2009). Considering these results it was unexpected that mutations in *CORONATINE INSENSITIVE1* (*COI1*), an essential component of JA perception, strongly increased resistance to *Focn* (Thatcher et al., 2009; Trusov et al., 2009). Importantly, loss-of-function mutations in additional regulators of JA signaling that are involved in pathogen defense, such as the transcriptional regulators MYC2, PFT1, and LBD20, also resulted in increased resistance to *Focn* (Anderson et al., 2004; Kidd et al., 2009; Thatcher et al., 2012b). These findings indicate that JA signaling-capacity of the host is critical for *Fo* pathogenesis in Arabidopsis.

Hijacking the host JA signaling machinery is a common strategy employed by many (hemi-)biotrophic pathogens and often involves suppression of SA-dependent defense responses (Pieterse et al., 2012; Kazan and Lyons, 2014). However, COI1 promotes *Fo* infection in an SA-independent manner as *coi1 NahG* plants remain as resistant as *coi1* plants (Thatcher et al., 2009). This indicates that JA signaling supports *Fo* pathogenesis by mechanisms other than antagonizing SA responses. Grafting experiments demonstrated that expression of *COI1* in roots, but not in shoots, is required for *Fo* infection (Thatcher et al., 2009). Cole and co-workers observed reduced colonization of the vasculature in *coi1* plants pointing to a role of JA signaling at relatively early stages (Cole et al., 2014). Thatcher et al. detected similar levels of fungal biomass in WT and *coi1* shoots before the switch to necrotrophic growth, and hence concluded that *COI1* is predominantly required for triggering plant decay (Thatcher et al., 2009). These differences might be explained by the inoculation methods used: uprooting of plants before inoculation could have created additional entry sites leading to stronger vascular colonization (as observed in Thatcher et al., 2009). Nevertheless, the studies indicate that COI1 influences the interaction with *Fo* at several stages. Moreover, the finding that even strongly colonized *coi1* plants remain essentially symptomless reveals an uncoupling of colonization and plant disease symptoms (Thatcher et al., 2009).

Despite its strong effects on the interaction with *Fo*, it is not well-understood how the host JA signaling machinery is re-wired by the pathogen to promote disease. Cole and co-workers found that two Arabidopsis-infecting strains, *Focn* and f.sp. *matthioli* (*Fomt*) produce JA-isoleucin and JA-leucin conjugates in culture filtrates that induce senescence-like symptoms on Arabidopsis leaves in a COI1-dependent manner (Cole et al., 2014). However, it is not yet known if these fungal derived hormones are also generated during infection, and to what extent they contribute to virulence. Alternatively, *Fo* effectors could play a role in JA signaling manipulation as has been suggested for the effector SIX4 (Thatcher et al., 2012a). It will be interesting to explore which JA signaling regulators are targeted by *Fo* and how their activity is modulated.

The dependency on host JA signaling for successful colonization however, is not conserved among all *Fo* ff.spp. For instance, *Forp* colonizes WT and *coi1* Arabidopsis plants to a similar extent and this isolate does not produce bioactive JA-conjugates *in vitro*. Similarly, *Fol* seems to infect tomato without JA-signaling manipulation as this f.sp. does not produce JAs and is not dependent on the tomato *COI1* homolog to cause disease (Cole et al., 2014). Thus, different *Fo* isolates have developed distinct infection strategies that either strongly rely on host JA signaling manipulation or involve alternative virulence mechanisms that are JA-independent.

In addition, several lines of evidence point to a role of JA in promoting resistance rather than susceptibility in plant species other than Arabidopsis. Tomato *def1* mutants that are defective in JA synthesis show enhanced susceptibility to *Fol*, which can be suppressed by exogenous JA treatment (Thaler et al., 2004). Similarly, *def1* tomato plants were more susceptible to root rot caused by *Fo* f.sp. *radicis-lycopersici* (*Forl*; Kavroulakis et al., 2007). Consistently, Sun and co-workers found that spraying of banana plants with Methyl-JA reduced disease incidence and severity caused by *Focb* Tropical Race 4 (Sun et al., 2013). In addition, resistant cultivars of strawberry and watermelon showed strong induction of the JA biosynthesis enzyme AOC upon inoculation with *Fo* f.sp. *fragariae* (Lu et al., 2011; Fang et al., 2013). A positive role of JA for defense activation was also found in date palms inoculated with *Fo* f.sp. *albenidis* (Jaiti et al., 2009).

In summary, JA signaling promotes defense to *Fo* in different plant species, but can also be hijacked to induce pathogenicity in at least Arabidopsis (Figure 1). Further research will be necessary to reveal via which mechanisms JA contributes to disease reduction or induction, and which function is predominant among different plant species.

## Dual role of ET in activation of both defense responses and disease symptoms

Generally, ET together with JA mediates the resistance response to necrotrophic pathogens. However, ET can also positively influence defense responses to hemi-biotrophs and induced systemic resistance, which is triggered by beneficial root-associated microbes (Pieterse et al., 2014; Broekgaarden et al., 2015).

Pre-treatment of Arabidopsis seedlings with either MeJA or the ET precursor 1-aminocyclopropane-1-carboxylic acid (ACC) resulted in enhanced disease symptoms upon *Focn* inoculation, indicating that both these hormones promote disease development (Trusov et al., 2009). Accordingly, the ET-insensitive Arabidopsis *ein2-1* and *etr1-1* mutants showed a reduction of disease symptoms compared to WT Col plants when inoculated with *Focn* or *Forp*, respectively (Table 2; Trusov et al., 2009; Pantelides et al., 2013). It is worth mentioning that enhanced disease upon MeJA treatment, as well as reduced disease in ET-insensitive plants was not observed in similar studies, indicating that these effects are either weak or depend on yet unknown factors (Edgar et al., 2006; Thatcher et al., 2009). Moreover, a different *ein2* allele (*ein2-5*) even showed markedly enhanced susceptibility to *Focn* under sterile conditions (Berrocal-Lobo and Molina, 2004). These findings point to an age- and/or condition-dependent role of ET in Arabidopsis interaction with *Fo*.

Global transcriptome profiling in Arabidopsis and banana plants inoculated with virulent *Fo* strains revealed a massive induction of ET/JA-responsive genes such as *Plant Defensins* (*PDF*s) and *Pathogenesis-Related* (*PR*) genes as well as genes encoding ethylene biosynthesis enzymes (McGrath et al., 2005; Kidd et al., 2011; Li et al., 2013; Zhu et al., 2013; Lyons et al., 2015). Furthermore, the transcriptome profiles indicated that initial activation of ET-dependent genes precedes the activation of JA, SA and ABA signaling (Li et al., 2013; Zhu et al., 2013). Altogether, these findings suggest a model in which initial ET/JA-associated defenses are mounted in response to *Fo* infection, but these are typically insufficient to stop the pathogen. At later stages however, ET signaling can rather enhance disease symptoms and possibly also pathogen proliferation. This hypothesis is further supported by the observation that Arabidopsis plants overexpressing certain *ERF* transcription factors, thereby constitutively activating ET/JA-dependent defense responses, become less susceptible to *Focn* (Berrocal-Lobo and Molina, 2004; McGrath et al., 2005). However, whether ET signaling is actively suppressed and/or at later stages co-opted by *Fo* to promote pathogenesis remains to be addressed.

The exact role of ET in other plant species is relatively little understood. The tomato *Never ripe* (*Nr*) mutant is impaired in ethylene perception and shows reduced disease symptoms upon *Fol* inoculation (Lund et al., 1998; Francia et al., 2007). Interestingly, previous work revealed a role for ET in mediating xylem occlusion through formation of gels in castor bean (Vandermolen et al., 1983). Xylem occlusion is thought to limit pathogen spread, but also to contribute to the typical wilting symptoms (Yadeta and Thomma, 2013). Thus, it is an interesting question whether *Nr* tomato plants allow systemic fungal spread and how this would correspond to the observed reduction in disease symptoms. Resistance to *Forl*, a pathogen which adopts a necrotroph-like lifestyle, was largely unaffected in two tested ET-insensitive tomato lines, *Nr* and *epinastic* (*epi*; Kavroulakis et al., 2007). However, protection mediated by an endophytic *Fusarium solani* strain was greatly reduced in *Nr* and *epi* tomato plants and hence required intact ET signaling (Kavroulakis et al., 2007).

In conclusion, the present studies underline a multifaceted role of ET signaling that strongly depends on the interaction stage, the host plant and environmental conditions (Figure 1).

## ABA promotes shoot disease symptoms but not root colonization in arabidopsis

Besides its well-described role in development and abiotic stress responses, ABA has been increasingly recognized as a critical regulator of biotic interactions. ABA can either positively or negatively influence resistance largely depending on the encountered pathogen (Ton et al., 2009; Robert-Seilaniantz et al., 2011).

Similarly to Methyl-JA and ACC, exogenous treatment with ABA increased Arabidopsis susceptibility to *Focn* (Trusov et al., 2009). Consistently, Arabidopsis mutants in which either ABA biosynthesis or signaling is disrupted showed fewer symptoms (Table 2; Anderson et al., 2004; Trusov et al., 2009). The reduced susceptibility of ABA mutants was associated with hyper-activation of ET/JA-dependent defense genes, likely due to antagonistic interactions between ABA and ET signaling (Anderson et al., 2004). ABA could also antagonize SA-dependent responses (Yasuda et al., 2008), but currently it is unknown if the interaction with other hormones explains reduced *Fo* symptoms in ABA-deficient mutants.

Interestingly, *Fo* successfully colonized the roots of ABA-deficient mutants to a similar extent as those from WT plants (Cole et al., 2014). This would point to a role of ABA during the switch to the necrotrophic phase. However, transcriptome profiling revealed activation of numerous ABA responsive genes in the roots of *Fo*-inoculated plants (Lyons et al., 2015). Previous studies indicated that ABA mediates root-to-shoot defense signaling in plants (Balmer et al., 2013). This raises the possibility that *Fo* co-opts systemic ABA signaling to manipulate root-shoot signaling. Moreover, it has been shown that, for example during defense against herbivorous insects, ABA signaling can serve to activate or enhance the MYC2-regulated branch of JA signaling (Kazan and Manners, 2013; Vos et al., 2013). However, if and how exactly *Fo* manipulates ABA signaling, is currently unknown.

## Auxins affects both root colonization and shoot symptom development

Auxins are major regulators of plant growth and development, but have also profound effects on interactions with both pathogenic and mutualistic microbes (Robert-Seilaniantz et al., 2011; Zamioudis et al., 2013).

Exogenous application of auxin or auxin biosynthesis inhibitors did not affect disease development in *Focn*-inoculated Arabidopsis (Kidd et al., 2011). Similarly, mutants with either reduced or increased auxin levels behaved like WT plants. In contrast, *Focn*-inoculated auxin-signaling mutants showed markedly reduced symptoms relative to WT plants (Table 2). Additionally, alteration of polar auxin transport, either by chemical inhibitors or in mutants, resulted in increased resistance to *Focn* (Kidd et al., 2011). These data indicate that local changes of auxin levels and/or distribution are important for disease susceptibility. Indeed, histological visualization of *DR5* expression, a well-known auxin reporter gene, revealed activation of auxin signaling at root tips and lateral root initials, two preferred *Fo* entry sites in Arabidopsis (Czymmek et al., 2007; Kidd et al., 2011; Diener, 2012). Additionally, Diener revealed that fewer root tips are colonized in plants mutated in the auxin efflux carrier *PIN2/EIR1* (Diener, 2012). In contrast, *tir3* mutants which are defective in polar auxin transport show 2–3 fold higher *Fo* biomass in roots, but disease symptoms of the shoot remained strongly reduced (Kidd et al., 2011; Diener, 2012). These findings suggest that auxin signaling and transport affect several stages of the *Fo*–Arabidopsis interaction from initial root tip colonization to disease symptom expression in the shoot. However, the mechanisms by which auxin promotes colonization and symptom development are still enigmatic. Previous studies describe an antagonistic relationship between auxin and SA signaling, however the *Fo* disease phenotypes of auxin signaling mutants were not SA-dependent (Kidd et al., 2011).

Auxin accumulation, transport, and signaling are modulated by numerous different symbiotic and pathogenic organisms including bacteria, fungi, nematodes, and even parasitic plants during their interaction with roots (Grunewald et al., 2009; Kazan and Manners, 2009; Zamioudis and Pieterse, 2012). This suggests that manipulation of the host auxin signaling pathway represents a common strategy employed by diverse root colonizers resulting in either detrimental or beneficial effects for plants.

## Are phytohormones determinants of *Fo* lifestyle?

Interestingly, changes in the phytohormone network can uncouple colonization by *Fo* from plant disease development. For instance, specific mutants with impaired JA, ABA, and auxin signaling still allow extensive root (and sometimes shoot) colonization but have greatly reduced disease symptoms (Thatcher et al., 2009; Diener, 2012; Cole et al., 2014). Similarly, resistant tomato plants that are completely free of symptoms can have their shoots and stems extensively colonized by *Fol* (Mes et al., 1999). Furthermore, a *Fol* knockout strain lacking the Six6 effector triggered vascular browning in a susceptible tomato cultivar, indicative of successful xylem colonization, but exerted almost no negative effects on plant growth and development (Gawehns et al., 2014). Altogether, these findings suggest that manipulation of plant hormone signaling rather than colonization triggers disease symptom development.

How does *Fo* manipulate the plant hormone network? One mechanism could be the production by the fungus of hormone-like secondary metabolites, including JAs, auxins, gibberellic acids, and ethylene (Hasan, 2002; Cole et al., 2014; Bitas et al., 2015). *Fo* also secretes numerous small proteins during plant infection, which might be another means to manipulate the host. For several of these proteins a virulence-promoting function has been shown designating them as effectors *sensu strictu* (Takken and Rep, 2010; de Sain and Rep, 2015). Among these, SIX4 was found to enhance JA signaling during infection of Arabidopsis (Thatcher et al., 2012a). Infection of tomato plants with *Fol* knockout strains lacking specific effectors revealed common and unique effects on the xylem proteome composition raising the possibility that each effector targets a distinct hormone signaling pathway (Gawehns et al., 2015). However, for the vast majority of *Fo* effectors their working mechanism remains unknown. It will be interesting to explore if plant hormone-synthesis or -signaling represents a recurrent virulence target of *Fo* strains on various hosts. Furthermore, it is tempting to speculate that at least a subset of the proteinacious effectors mediate immune suppression enabling (endophytic) colonization during the biotrophic phase of infection, whereas secondary metabolites with hormonal- or toxic-activity trigger plant damage/death during necrotrophic growth.

A growing body of evidence suggests that the majority of *Fo* strains survive in soil, in the rhizosphere or within plant tissues without causing disease symptoms, and some strains even confer extensive beneficial effects (Alabouvette et al., 2009; Edel-Hermann et al., 2015; Imazaki and Kadota, 2015). The existence of such a widespread and intimate co-habitation points to a finely balanced interaction between plant and fungus. The finding that also pathogenic strains can reside inside plant tissues without damaging the host indicate that one *Fo* isolate can employ diverse interaction/colonization strategies whose outcome possibly depends on the “compatibility” of a putative host plant. Thus, the “infection tools” of a *Fo* strain, likely comprised of a combination of effectors, enzymes, and secondary metabolites, determine the outcome of an interaction: either endophytic with potential beneficial effects for the host or pathogenic with various levels of disease and in extreme cases plant death. This idea is supported by the observation that transfer of one specific *Fol* chromosome, which contains most of its effector genes plus a secondary metabolite cluster, conferred pathogenicity to an endophytic strain (Ma et al., 2010).

Clearly more research in the areas of genomics and effector biology is required to understand how *Fo* manages to trick the hormonal network of its hosts, and how these interactions can have opposite outcomes ranging from pathogenesis to mutualism.

## Conclusions and outlook

Phytohormones determine colonization and disease symptom development during interactions with pathogenic *Fo* strains. However, their roles vary depending on the host plant and the fungal strain involved, suggesting that the manipulation of the host hormonal network differs between individual *Fo* strains. Genetic interference with hormone regulators mostly reduced disease symptoms, as seen for JA, ABA, and auxin, indicating that the ability to hijack plant hormone pathways is a requirement for pathogenesis. This scenario implies a strong adaptation to a particular host plant, potentially leading to the narrow host range observed in the *Fo* species complex. How exactly the manipulation of phytohormone signaling differs between *Fo* strains—and if this is indeed the key difference between pathogenic and endophytic interactions—remains an intriguing question for future research. Comparison of the respective effector repertoires as well as a better understanding of their mode of action will help answering these questions and may furthermore reveal novel approaches for plant protection, either by breeding or by optimizing *Fo* strains for biocontrol.

## Author contributions

XD and NT wrote the manuscript together with input from FT. The scope and the topic were developed by XD, NT, and FT.

### Conflict of interest statement

The authors declare that the research was conducted in the absence of any commercial or financial relationships that could be construed as a potential conflict of interest.
